# Effective Adoption of Tablets for Psychodiagnostic Assessments in Rural Burundi: Evidence for the Usability and Validity of Mobile Technology in the Example of Differentiating Symptom Profiles in AMISOM Soldiers 1 Year After Deployment

**DOI:** 10.3389/fpubh.2021.490604

**Published:** 2021-04-15

**Authors:** Roland Weierstall, Anselm Crombach, Corina Nandi, Manassé Bambonyé, Thomas Probst, Rüdiger Pryss

**Affiliations:** ^1^Department of Psychology, Medical School Hamburg, Hamburg, Germany; ^2^Department of Psychology, University of Konstanz, Konstanz, Germany; ^3^Department of Clinical Psychology, University Lumière de Bujumbura, Bujumbura, Burundi; ^4^Department for Psychotherapy and Biopsychosocial Health, Danube University Krems, Krems, LA, Austria; ^5^Institute of Clinical Epidemiology and Biometry, University of Würzburg, Würzburg, Germany

**Keywords:** tablet computer, application, post-deployment aggression, PTSD, depression, latent-profile-analysis, soldiers, mobile data collection

## Abstract

Research on the use of mobile technology in health sciences has identified several advantages of so-called mHealth (mobile health) applications. Tablet-supported clinical assessments are becoming more and more prominent in clinical applications, even in low-income countries. The present study used tablet computers for assessments of clinical symptom profiles in a sample of Burundian AMISOM soldiers (i.e., African Union Mission to Somalia; a mission approved by the UN). The study aimed to demonstrate the feasibility of mHealth-supported assessments in field research in Burundi. The study was conducted in a resource-poor setting, in which tablet computers are predestined to gather data in an efficient and reliable manner. The overall goal was to prove the validity of the obtained data as well as the feasibility of the chosen study setting. Four hundred sixty-three soldiers of the AMISOM forces were investigated after return from a 1-year military mission in Somalia. Symptoms of posttraumatic stress disorder (PTSD) and depression were assessed. The used data-driven approach based on a latent profile analysis revealed the following four distinct groups, which are based on the soldiers' PTSD and depression symptom profiles: Class 1: moderate PTSD, Class 2: moderate depression, Class 3: low overall symptoms, and Class 4: high overall symptoms. Overall, the four identified classes of soldiers differed significantly in their PTSD and depression scores. The study clearly demonstrates that tablet-supported assessments can provide a useful application of mobile technology in large-scale studies, especially in resource-poor settings. Based on the data collected for the study at hand, it was possible to differentiate different sub-groups of soldiers with distinct symptom profiles, proving the statistical validity of the gathered data. Finally, advantages and challenges for the application of mobile technology in a resource-poor setting are outlined and discussed.

## Introduction

In recent years, more and more studies and meta-analyses have investigated and shown the usefulness of mobile technology in psychological research, clinical assessments, and therapeutic interventions [e.g., (1, 2)]. In particular, digital assessments and interventions offer several advantages but also challenges [e.g., (3, 4)]. On the one hand, the collection of data can be improved by reducing time efforts and resources, especially when large data sets have to be processed in a rather short period of time (5). On the other hand, a suitable infrastructure has to be provided, additional development costs have to be covered, and data security issues must be addressed [e.g., (6, 7)].

However, the implementation of mobile health (mHealth) applications is not solely bound to practical issues. The validity of the gathered data via mobile devices has to be considered carefully. As the proliferation of mobile technology has increased by orders of magnitude, more and more researchers address validity issues in the context of mHealth-collected data. In the beginning of the development of mHealth applications, no guidelines existed on how interventions based on mHealth have to be reported in scientific applications, making it difficult to compare the quality of research designs. The World Health Organization (WHO) mHealth Technical Evidence Review Group therefore developed the mHealth evidence reporting and assessment (mERA) checklist in 2016, covering 16 items to be addressed when reporting mHealth applications in scientific publications (8).

Although extant research on mHealth mostly stems from western countries, several studies in resource-poor countries have emerged in recent years that used mobile technology; e.g., in the East African countries Burundi, Rwanda, and Uganda [e.g., (9–11)]. The latter studies covered aspects on interventions as well as diagnostic questions. This demonstrates that mHealth does not have to be limited to studies in first-world countries, but can be applied all over the world. The validity of the psychodiagnostic assessments, however, is a substantial and necessary prerequisite for any further clinical research for example on interventions based on mHealth applications and mobile technology. Therefore, the use of mobile technology is demonstrated in this paper based on psychodiagnostic assessments in a sample of Burundian soldiers. The conducted study utilized tablet computers to carry out standardized clinical interviews digitally. Instead of solely focusing on the users' willingness to use a mobile application, this paper aims to demonstrate - on the basis of a data-driven approach - that the gathered data is valid and suitable for further clinical research questions.

There is a considerable amount of public attention on psychological consequences of mental health problems in soldiers in the aftermath of their deployment (12, 13). Among the various disorders reported in the literature, the post-traumatic-stress disorder (PTSD) and depression are among the most common ones (14). While some studies primarily focus on either depression (15) or PTSD symptoms (16), others acknowledge the high comorbidity between the two disorders (17). Although the relation between PTSD and depression in soldiers after deployment is still under debate (18), it is reasonable to assume that different subgroups of soldiers exist. In these subgroups, in turn, soldiers might either display predominant PTSD or depression symptoms, or suffer from both of them (comorbidity). The present study therefore investigated whether or not distinct depression and PTSD symptom profiles can be identified in AMISOM (African Union Mission to Somalia) soldiers, based on a tablet-supported data collection solution.

The AMISOM (19, 20) is a UN Security Council authorized peacekeeping mission, sending military troops based in Uganda, Kenya, Burundi, Djibouti, and Ethiopia to stabilize Somalia and try to reclaim territories from the Al-Shabaab militia. AMISOM troops frequently cope with attacks and armed fights in a hostile environment. Beside mission related hassles, most Burundian soldiers experience a civil war that already started in 1993 and lasted more than a decade (21–24). During this period, the currently active Burundian soldiers often must fight against each other, including fights with former government troops or rebel movements. As a consequence of these particular circumstances, the affected population is challenged with severe traumas. Therefore, it is promising – and required - to investigate trauma- and deployment related mental-health consequences in these people.

The aim of this study was not only to demonstrate the general feasibility of structured clinical interviews in a low-income country that are accomplished through the use of tablet computers. On top of that, privacy concerns are discussed (25), which might have an impact on the participation in tablet computer guided interviews, leading to a general tendency to either disclose information or producing meaningless or invalid data. As an approach to validate the experts' ratings conducted in this study, it was tested, whether analyses of the collected data are capable to differentiate distinct symptom profiles in PTSD and depression symptoms. Altogether, a sample of 463 participants from the AMISOM was investigated 1 year after their deployment. A latent profile analyses (LPA) was conducted to separate different symptom profile groups. We expected to identify distinct diagnostic groups according to PTSD and depression.

## Materials and Methods

### Participants

The tabled-based diagnostic procedure was accomplished in a larger project aiming to improve the mental health status of Burundian soldiers of the AMISOM mission [for further details on PTSD rates and specific types of trauma-exposure pre- and peri-deployment see (17)]. The composition of the survey was limited to PTSD and depression as main psychiatric disorders and was primarily concerned with the assessment of deployment-associated risk factors. In general, this sample was exposed to various traumatic pre-deployment events [Median = 11 (17)], many of them thereby related to the Burundian civil war. Many of the sample have faced traumatic incidents during their deployment [Median = 5 (17)], including being attacked by an enemy, experiencing suicide attack, or witnessing comrades being killed.

For the present analysis, only full data sets of 463 participants were included who had been assessed 1 year after returning from their AMISOM deployment. No data imputation method was used in order to avoid any bias due to modeling data prior to the actual data-driven analysis. All participants were male. Mean age was 35 (*SD* = 5 years) at the time of interview (i.e., post deployment). Out of the 463 soldiers, 81% reported to be married, whereas the rest was not in a stable relationship. Seventy-nine percentage of the soldiers had at least one child. On average, the soldiers had received 6 years of formal education (*SD* = 2 years). Out of the 463 soldiers who reported their military rank, the following main ranks were obtained: major: corporal: 219 (48%); chief corporal: 173 (38%); other: 66 (14%).

The participation was on a voluntary basis. Participants received no incentives for participation. All participants gave written informed consent. Oral informed consents were collected in case of illiteracy. The Ethical Review Boards of the University of Konstanz and the University of Bujumbura, Burundi approved the study. The study was conducted in cooperation with the Force de la Défense National, Burundi. All parties involved granted strict confidentiality, acknowledging the specific vulnerability of the target population.

### Study Procedure

Clinical symptoms were assessed using standardized clinical interviews, guided by a survey implemented for tablet computers (i.e., Apple ipads). All participants were released from their routine duty for the interviews. To ensure anonymity and confidentiality, electronic coding and storage of the data was utilized, which fulfilled the highest and most secure data encryption standards (7). Before their application in the interviews, all questionnaires had been translated into Kirundi, using back and forth translations (26). Trained mental health experts from Burundi and Germany conducted all interviews in Kirundi, so that literacy was not an issue. Bi-lingual local interpreters supported the German mental health experts. All questionnaires were translated from English into Kirundi using back-and-forth translations. All translations were discussed in an experts' panel consisting of bi-lingual translators as well as mental health experts from Burundi and Germany. Assessments were conducted in different military camps of the Burundian army and lasted about 2 h. Separate barracks were provided for the implementation of the research project by the Burundian army and interviews were conducted individually to prevent any undue influence or the issue of stigma, which could have resulted from group-based assessments. Interviewers entered the participants' responses into the iPad and probed the responses prior to the rating.

All questionnaires and scales were administered on tablet computers, using the software and technical equipment described below. Only written informed consent was collected by paper-pencil mode. Interviews were carried out in a private space between the participant, the clinical interviewer, and if necessary, by a local interpreter. Clinical interviewers had to rate symptoms and responses using the tablet computers. Experienced international and local clinical psychologists, and Burundian psychology students, who had been – just like the interpreters - excessively trained in mental health concepts, were continuously supervised during the assessment period, and carried out the diagnostic interviews. Ongoing intervision and rotating supervisors, which attended the interviews at random, ensured a high quality of the interviews.

### Assessment of Posttraumatic Stress Symptoms

The fifth version of the PTSD Symptom Scale Interview [PSS-I; (27)] was administered for the assessment of PTSD symptoms. It is a 20-item interview that assesses each symptom of the DSM-5 during the past month for severity and frequency. However, due to the necessity to keep the results comparable to previous assessments, the response options for each item ranged in accordance with the DSM-IV version on a four-point Likert scale from 0 (not at all) to 3 (five or more times per week/almost always), instead of the newly adapted five-point Likert scale of the DSM-5 version. The PSS-I has proven its validity already in an application with soldiers from the Burundian Army prior to their deployment and in a sample of former Burundian combatants (28). Homogeneity in the present sample was satisfying (Cronbach's Alpha = 0.89). To conduct latent profile analysis (LPA), individual item scores were used. To distinguish class profiles, sub-scores for the DSM B (PSS-I re-experiencing), C (avoidance), D (PSS-I negative changes in cognition and mood) and E (PSS-I increased arousal and reactivity), criteria across classes were compared. Mean PSS-I score in the present study was 4.4 (*SD* = 6.1); clusters: reexperiencing (*M* = 1.1, *SD* = 2.0), avoidance (*M* = 0.6, *SD* = 1.0), negative changes in cognition and mood (*M* = 1.1, *SD* = 2.1), and increased arousal and reactivity (*M* = 1.3, *SD* = 2.1).

### Assessment of Depression Symptoms

Depression symptoms were assessed with the Patient Health Questionnaire-9 (PHQ-9), a well validated, and short severity measure of depression [cf. (29)]. The PHQ-9 was originally designed as a self-rating instrument, but has been successfully implemented in clinical interviews as well (14). For the identification of symptom profiles in the LPA, individual items were included in the analysis. However, for the specification of class characteristics, class differences in the PHQ-9 sum score were analyzed. Therefore, the item scores for the assessment of symptom frequency were summed up (*M* = 2.7, *SD* = 3.7). Cronbach's Alpha for the entire scale was 0.85.

### Mobile Devices

The data collection procedure was performed using mobile devices. As a new iOS tablet application with particular characteristics was developed for this study, some aspects of more general interest are shortly discussed. The overall time to develop the mobile application was rather tight (i.e., roughly 8 weeks), therefore an approach from computer science was chosen that is called rapid prototyping [cf. (30)]. However, only using this well-known approach was not sufficient enough in the end, more ideas had to be created and technically carried out to cope with the challenges of the study. As it turned out that the application had to be changed frequently on-site, having in mind that often no internet connection is available and the computer scientists were not present in Africa, a procedure had to be found to transfer application changes from Germany to Burundi. The reasons for these change demands were mainly due to language issues, new interview functions (e.g., feature to quickly jot down notes), or user interface changes. Beside on-site changes, it was challenging to cope with requirements pertaining to the provided procedure how questionnaires are filled out by the psychologists. They wanted features to navigate through questionnaires that required to implement individual features by the computer scientists. In the light of the short implementation time, while preserving validity and integrity of the collected data at the same time, the implementation phase was challenging before the study as well as during the study. As another important aspect, the procedure how data was stored on the used iPads as well as securely transferred for statistical analyzes, was also challenging and required new ideas, procedures, and features. This included implementation efforts as well as training efforts between the psychologists and the computer scientists. Finally, note that the mobile application was not installed to the used iPads using the official App Store from Apple. Instead, the mobile application was directly installed to the iPads; i.e., before the interviewers left Germany to Burundi.

### Mobile Data Assessment and Data Security

The collection procedure for the study at hand was accomplished using the aforementioned mobile application. At the time of the study, 3rd generation iPads have been used. During the collection procedure, three aspects were particularly relevant. First, all data must be locally stored on the iPads to properly consider the local circumstances. In addition, data must be locally secured. For this purpose, data was anonymized and encrypted. For encryption purposes, the AES-256 encryption algorithm was used (31). Second, a multi-user feature was implemented to distinguish between interviewers and administrators. The latter were the only entitled persons to decrypt all locally store data. Therefore, administrators had their own area within the mobile application, which was also secured with a password. To secure the data transfer procedure even more strictly, the data transfer was only possible from the iPads to a stationary PC in this administrator area using iTunes. Third, applications adaptions during the data collection procedure became actually necessary. Technically, if adaptions had to be carried out, they were accomplished using an SVN server (32) to which the psychologists in Burundi had remote access. To properly use this access method, the psychologists were taught by the computer scientists in Germany how to deploy a new version of the mobile application. The final collected and anonymized data was secured according to data protection regulations in Germany and stored for 10 years.

### Data Analysis

The data analysis was conducted in two steps. First, a latent profile analysis (LPA) (33) was performed to identify subgroups of soldiers based on the PHQ-9 and PSS-I items, accounting for depression and PTSD symptoms simultaneously. The LPA was conducted using Mplus 7 for Mac. A LPA uses latent categorical variables to identify groups of individuals with similar symptom patterns (classes) on a set of clinical variables. In comparison to other statistical approaches that aim to identify groups of participants within a dataset, like a cluster analysis, LPA has several advantages, in particular the “availability of more rigorous empirical criteria for determining the number of clusters” (34). Due to positively skewed, over-dispersed, and non-normally distributed outcome data, a negative binomial model was preferred over linear or Poisson regression models. Applying a zero-inflated negative binomial model to the data was discarded due to unacceptable fit indices. For the appropriate assignment of class labels, one-way analyses of variance (ANOVAs) were conducted with group membership as the independent variable and depression as well as PTSD symptom severity as dependent variables. For the selection of the appropriate number of classes, Lo–Mendell–Rubin-adjusted likelihood ratio tests (LMR-A) as well as bootstrap likelihood ratio test (BLRT) were calculated, indicating the superiority of the final model in comparison to models with a different number of classes [cf. (33)]. Additionally, the Bayesian Information Criterion (BIC) was chosen as a model fit indicator. Analyses were conducted using R statistics, applying a cutoff-level for significance of *p* = 0.05.

## Results

### Class Assignment by Symptom Profiles

**Figure 1:**
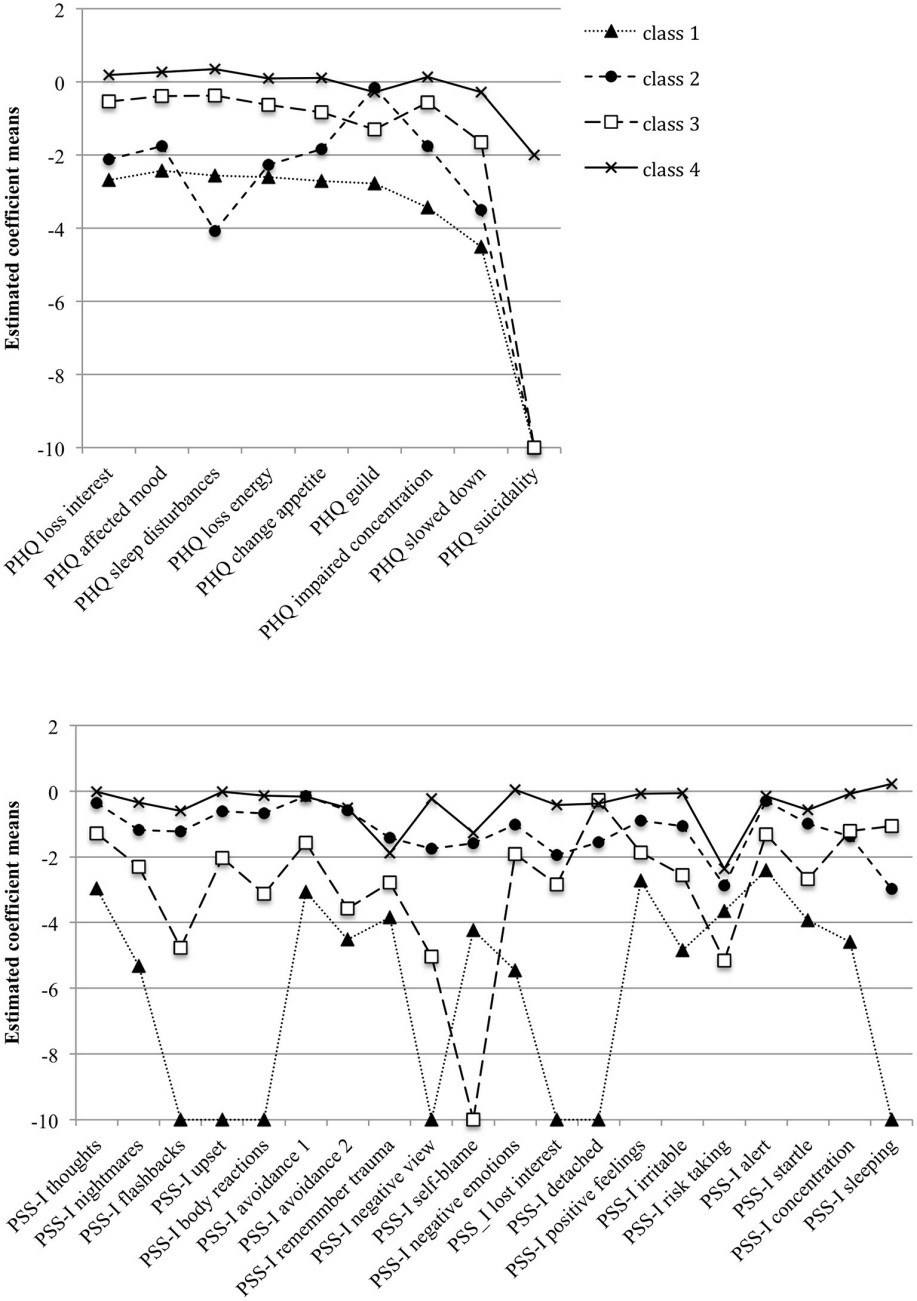
Estimated coefficient means in the PHQ-9 and PSS-I items across the four classes derived from the Latent Profile Analyses. For a better visual presentation, coefficients smaller than−10 were set to−10.

In a first step, latent profile analyses were calculated for two- to eight-classes models, using negative binomial models with automatic starting values and random starts. For the two-class model, LMR-A and BLRT tests were significant on a *p* < 0.05 level. For the comparison between the three- and four classes model, the LMR-A test did not reach statistical significance. However, the BIC value favored a four classes model. Thus, according to the recommendations by the authors of (10), and in line with the results demonstrating that models with more than five classes did not further improve the model fit, the four-class model was selected for all further analyses on the identification of latent profiles (Cluster 1: *n* = 194, 41.9 %; Cluster 2: *n* = 91, 19.7 %; Cluster 3: *n* = 115, 24.8 %; Cluster 4: *n* = 63, 13.6 %). Table 1 and Figure 1 give an overview and illustration of the seven different models' fit indices.

**Table 1:** Fit indices for the seven different latent profile analyses.

**Modell**	**Log-likelihood**	**BIC**	**Entropy**	**LMR-A *p***	**BLRT *p***
2 classes	−7,828.89	14,184.64	0.914	<0.001	<0.001
3 classes	−6,826.47	13,905.54	0.895	0.232	0.231
**4 classes**	–**6,591.97**	**13,762.56**	**0.881**	**0.103**	**0.103**
5 classes	−6,428.32	13,818.59	0.888	0.502	0.503
6 classes	−6,396.03	13,981.80	0.891	0.735	0.736
7 classes	−6,352.41	14,173.28	0.901	0.655	0.656
8 classes	−6,306.72	14,258.65	0.911	0.340	0.340

*BIC, bayesian information criterion; LMRA-A, Lo-Mendell-Rubin adjusted likelihood ratio test; BLRT, bootstrap likelihood ratio test. The bold values represent the finally selected model*.

Results in Table 2, in turn, indicate a number of cases in each of the four classes between 63 and 194 participants. The posterior probabilities that participants belonged to their assigned class ranged between 0.89 and 0.99. Therefore, the model selection produced a meaningful class assignment with four distinguishable classes.

**Table 2:** Average posterior probabilities for the 4-class model.

**class**	**N**	**1**	**2**	**3**	**4**
class 1	194	**0.95**	0.01	0.04	0.00
class 2	91	0.02	**0.93**	0.04	0.02
class 3	115	0.05	0.04	**0.89**	0.02
class 4	63	0.00	<0.01	<0.01	**0.99**

Posterior probabilities represent the probability that an individual belongs to the respective assigned class. The bold values highlight the correct classification.

### Differences in Psychopathology Between Classes

**Figure 2:**
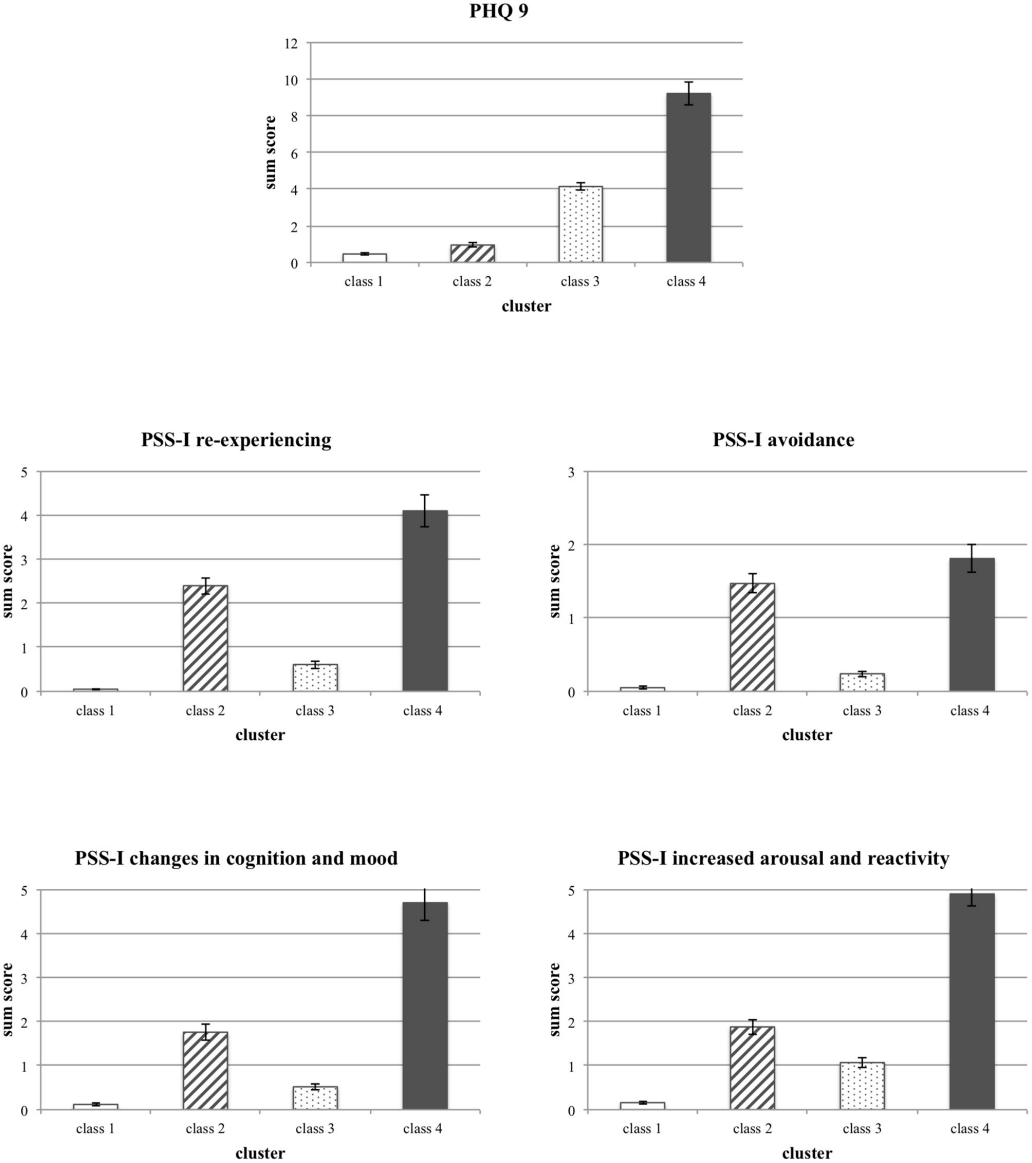
Differences in the mean scores between the four classes of participants for the PHQ-9 sum score as well as the PSS-I sub-scales. Means and Standard Deviations are displayed.

In a second step (for a better illustration, see Figure 2), mean differences between classes were compared using one-way ANOVAs for the (1) PHQ-9 scale [*F*(3, 459) = 291.50, *p* < 0.001, η*p2* =0.66], the (2) PSS-I re-experiencing scale [*F*(3, 459) = 161,19, *p* < 0.001, η*p*2 = 0.51], the (3) PSS-I avoidance scale [*F*(3, 459) = 115.23, *p* < 0.001, η*p2* = 0.43], the (4) PSS-I changes in mood and cognition scale [*F*(3, 459) = 161.64, *p* < 0.001, η*p2* = 0.51], and the (5) PSS-I hyper-arousal scale accordingly [*F*(3, 459) = 215.04, *p* < 0.001 η*p2* = 0.58]. Almost all *post-hoc* Tamhane t2 tests for pairwise comparisons were also statistically significant (all *p* < 0.001), except for the difference between (1) PHQ-9: Class 1 vs. Class 2, (2) PSS-I avoidance scale: Class 1 vs. Class 3 and Class 2 vs. Class 4, and (3) PSS-I changes in mood and cognition scale: Class 1 vs. Class 3.

Thus, the results revealed four classes with distinct symptom profiles. Class 1 (low overall symptoms) had the lowest scores on all mental health measures, whereas Class 4 (high overall symptoms) had the highest symptoms scores on both, depression and PTSD scales. Participants in Class 2 (moderate PTSD) showed moderate PTSD symptoms, and in comparison, to the other three groups, low to almost no depression symptoms. Class 3 (moderate depression) was characterized by moderate depression symptoms and low to almost no PTSD symptoms.

## Discussion

The results of this study emphasize the feasibility of tablet-assisted clinical interviews assessing mental health symptoms in resource-poor post-conflict regions, such as Burundi. Using a latent class analysis, we identified four different symptom cluster groups amongst Burundian soldiers in a 1-year follow-up after the deployment in AMISOM. Those cluster groups included a low overall symptom profile, a moderate PTSD profile, a moderate depression profile, and within a minority, a high overall-symptom profile.

These findings indicate that mental health symptoms related to depression and PTSD are clustered in a similar way as we would expect amongst soldiers from high-income countries after deployment (35, 39–42). This result could indicate that mental health symptoms as a reaction to traumatic and/or daily stressors might be indeed similar between different cultures (13). The results add to the mounting evidence that mental health concepts and assessment tools developed in high-income cultures can be successfully adapted to different cultural backgrounds (13). These implications would be in line with evidence suggesting that many symptoms of trauma-related disorders and also depressive symptoms result from universal physiological reactions to stress, e.g., the ways traumatic memories are processed within the brain (36, 37).

The majority of the participants had little to no experience with tablets prior to this mental health project, and many of them had little school education. Nevertheless, the use of tablets in the clinical interviews seems not to have affected their willingness to talk about their mental health problems with clinical mental health experts in a way, which provides meaningful results. This conclusion is very promising and might allow researchers and mental health services in resource-poor countries to use mobile technology for meaningful assessments and service provisions. As technology will get more accessible and its' use more usual in every culture in the future, overcoming possible remaining obstacles, such as unfamiliarity with use for self-assessments, and/or lack of alphabetisation, seems very likely.

However, while we could identify four clearly distinct symptom profiles, the mental health symptoms were not that pronounced in individuals of our sample. Taking the mean values of PTSD symptoms (*M* = 4.4) and depression symptoms (*M* = 2.7) into account, we have to acknowledge that we either assessed a highly resilient group of individuals, the majority of whom did not develop severe mental symptoms despite their significant exposure to traumatic stress and violence, or that the soldiers underreported some of their mental health symptoms. Most likely, both of these potential explanations contribute to the low reported symptom scores. The soldiers we assessed remained in the Burundian army after the end of the civil war, when many of those severely affected by injuries had been demobilized. The soldiers continuously took benefit from unit support and relative income stability resulting from their status. Furthermore, they have been less exposed to traumatic experiences than their demobilized colleagues, and might have been particularly adapted to traumatic and violent environments (28). However, mental health problems are also associated with stigma, particularly amongst soldiers. For some of the soldiers, symptoms might be underreported due to the necessity being regarded as strong and functional soldiers, and to avoid any risk of demobilization. Even though, we informed them that no individual information would be passed on to superiors, nevertheless, comprehensibly, a certain mistrust remained. The fact that we successfully identified the four symptoms clusters despite the low symptom Scores, might indicate that those symptoms have been in fact underreported. Hence, sophisticated statistical methods, such as latent class analysis, help to better understand underlying properties of data that would otherwise not be detectable, thereby confirming reliability and validity of the data gathered by the use of mobile technology

Other analyses methods could be applied to expand the results of the latent class analysis. For example, machine-learning methods could be used to re-evaluate the classes. Based on the number of variables and participants, several machine learning approaches could be a valuable target (e.g., support vector machines). One limitation of this study is that professionals interviewed and entered the data for the soldiers. In consequence, participants might have been less open to report about their mental health symptoms, as research from high-income countries indicates that participants generally tend to be more honest when providing their answers directly to a digital solution [cf. (38)]. Possibly, this circumstance could be improved in the future with more literate samples, although it remains unclear if the reporting bias identified within high-income countries toward more openness is the same within cultures less exposed to technology when reporting health issues to a machine. Another shortcoming relates to the fact that the same measures were used for identifying and validating the profiles. After a set of PTSD and depression measures is used to empirically determine profiles, using a new set of PTSD and depression measures to validate the profiles would provide a stronger validation approach.

However, it was striking during this study that the mobile application has several benefits compared to a traditional paper-based study. A higher amount of collected data in a rather short time and with higher data quality could be achieved. Regarding the data quality, transcription errors are minimized since a procedure to digitize the data is no longer necessary. In addition, by easily switching between different languages for a questionnaire, the collection procedure could be also improved since translation issues could be mitigated. For example, a psychologist can always toggle between languages if needed, which eases the understanding of questions at hand. Finally, being a challenge from the software engineering perspective, mobile applications that are used for studies must ensure that even when changes are applied to the implementation, the study results must be still valid and comparable. Therefore, data sets must be always tagged with a questionnaire version if substantial changes have been applied to the structure of the questionnaire or the general app implementation.

## Conclusion

With the present study, it could be demonstrated that mobile technology can enable clinical studies in a new, reliable, and innovative way, especially when studies are carried out in challenging environments. In particular, studies can be conducted in a rather short time with many advantages compared to traditional paper-based studies. For example, by gathering larger amounts of data and with less required resources when using tablet computers or smartphones. In addition, the application of recently emerging analysis methods like machine learning become more easily possible. This study has demonstrated that mobile technology is able to produce data sets, which are valuable and feasible for innovative analysis methods. However, the use of mobile technology also causes challenges that must be considered carefully. As this study showed that the implemented mobile application for the Apple iPad is able to reveal new and valuable research insights in the context of a large-scale study in a resource-poor setting, the general use of mobile technology for clinical studies, especially in challenging environments and with large-scale demands, seems to be promising.

## Data Availability Statement

The datasets generated for this study are available on request to the corresponding author.

## Ethics Statement

The studies involving human participants were reviewed and approved by Ethical Review Boards of the University of Konstanz and the University of Bujumbura, Burundi. The patients/participants provided their written informed consent to participate in this study.

## Author Contributions

RW, AC, and RP wrote the manuscript. RW, AC, CN, and MB defined the study design, were responsible for the data collection, and led the project. RW and AC conducted the data analysis and the pre-processing. RP developed the technical solution. CN, MB, and TP provided critical feedback on the manuscript. All authors contributed to the article and approved the submitted version.

## Conflict of Interest

The authors declare that this study received funding from the Volkswagen Foundation. The funder was not involved in the study design, collection, analysis, interpretation of data, the writing of this article or the decision to submit it for publication.
